# Deep learning segmentation with uncertainty quantification for spinal tuberculosis on fat-suppressed T2-weighted MRI

**DOI:** 10.3389/fphys.2026.1885886

**Published:** 2026-07-29

**Authors:** Xingyu Duan, Jiaxing Wang, Linan Wang, Yichao Fan, Jiong Wang, Qianpeng Ma, Ningkui Niu

**Affiliations:** 1 Department of Orthopedics, General Hospital of Ningxia Medical University, Yinchuan, Ningxia, China; 2 First Clinical Medical College, Ningxia Medical University, Yinchuan, Ningxia, China; 3 Department of Orthopedics, Zhoukou Central Hospital, Zhoukou, Henan, China; 4 Department of Orthopedics, The Third Hospital of Shizuishan (Pingluo People’s Hospital), Shizuishan, Ningxia, China; 5 Ningxia Key Laboratory of Clinical Pathogenic Microorganisms, General Hospital of Ningxia Medical University, Yinchuan, Ningxia, China

**Keywords:** deep learning segmentation, fat-suppressed T2-weighted MRI, physician validation, spinal tuberculosis, uncertainty quantification

## Abstract

**Background:**

Spinal tuberculosis is the most common extrapulmonary manifestation of tuberculosis. Magnetic resonance imaging (MRI), particularly fat-suppressed T2-weighted imaging (FS-T2WI), is the modality of choice for preoperative evaluation; yet indistinct lesion boundaries render manual delineation subjective and poorly reproducible. Although deep learning segmentation has advanced considerably, its “black-box” nature and limited interpretability remain critical obstacles. This study aimed to develop an uncertainty-guided deep learning segmentation framework and evaluate its accuracy and clinical utility.

**Methods:**

We retrospectively enrolled 210 patients with spinal tuberculosis from an initial cohort of 300 screened at three centers, and acquired preoperative FS-T2WI scans. Data from Center 1 (*n* = 160) were used for five-fold cross-validation, while the remaining 50 cases served as an external test set. We built an improved model on nnU-Net by integrating boundary-aware loss with Monte Carlo Dropout, and compared it against U-Net, Attention U-Net, and TransUNet. The uncertainty threshold was determined through internal cross-validation, and clinical validation followed a within-subject crossover design involving nine physicians.

**Results:**

On the external test set, the improved model achieved a Dice similarity coefficient of 0.858 and an AUC of 0.912, outperforming all comparative models. Uncertainty correlated strongly and positively with pixel-level error rates (Spearman *ρ* = 0.74). At a threshold of 0.52, sensitivity for identifying unreliable segmentation was 84.3% and negative predictive value was 89.5%. In the physician validation, overlaying uncertainty maps significantly increased trust scores (3.8 ± 0.7 vs. 3.2 ± 0.8; *P* < 0.001, Cohen’s *d* = 0.80) and reduced review time (*P* = 0.018), with the greatest benefit observed among resident physicians. In cases of obvious AI failure, uncertainty alerts accurately flagged erroneous regions in 80.0% of instances.

**Conclusion:**

The proposed uncertainty-guided framework improved spinal tuberculosis lesion segmentation accuracy. Pixel-level uncertainty maps reliably identified unreliable predictions and enhanced clinician trust, offering a robust AI decision-support tool for precise surgical planning.

## 1 Introduction

Spinal tuberculosis, the most common manifestation of extrapulmonary tuberculosis, frequently leads to vertebral destruction, kyphotic deformity, and neurological deficits, substantially compromising quality of life. Early and precise delineation of lesion extent—including the anatomical boundaries of vertebral destruction and paravertebral abscesses—is essential for surgical planning, defining the scope of debridement, and guiding anti-tuberculosis chemotherapy (Zhang et al., 2023; Jain et al., 2020; Ruparel et al., 2022). Because of its excellent soft-tissue contrast, magnetic resonance imaging (MRI) has become the preferred modality for diagnosing spinal tuberculosis and performing preoperative assessment. Among MRI sequences, fat-suppressed T2-weighted imaging (FS-T2WI) renders edema and inflammatory infiltration markedly hyperintense, and is particularly sensitive for detecting early vertebral involvement and paravertebral soft-tissue abscesses (Gao et al., 2017; Siddiqui et al., 2018). Nevertheless, spinal tuberculosis lesions often overlap with normal bone marrow edema, degenerative changes, or postoperative reactive alterations, and frequently exhibit blurred, irregular boundaries. Conventional manual slice-by-slice delineation is labor-intensive, highly dependent on physician experience, subjective, and poorly reproducible—limitations that render it inadequate for large-scale multicenter research and precision surgical planning.

In recent years, deep learning segmentation models such as nnU-Net have advanced considerably in medical image analysis (Isensee et al., 2021; Chang et al., 2025; van der Graaf et al., 2024); however, their application to spinal tuberculosis lesion segmentation remains challenging for two principal reasons. First, the low contrast between lesions and surrounding tissues, together with high inter-class similarity, makes models prone to false positives and false negatives at boundaries. Second, most existing deep learning models function as “black boxes,” providing no indication to clinicians of when or where predictions may be unreliable. This lack of interpretability severely limits the safe deployment of AI-assisted diagnostic systems in clinical practice (Ahmad et al., 2026; Lee et al., 2025; Qu et al., 2022). Uncertainty quantification techniques—particularly Monte Carlo Dropout (Gal and Ghahramani, 2015)—can estimate predictive confidence at the pixel level, visually flagging potentially erroneous regions and thereby transforming “unknown errors” into “identifiable risks” (Seoni et al., 2023; Garavelli et al., 2022). Most relevant studies, however, remain confined to technical performance validation, without rigorously designed clinician experiments to assess how uncertainty information truly influences clinical decision-making. Therefore, this study aimed to develop an uncertainty-guided deep learning segmentation framework that incorporates boundary-aware loss to optimize accuracy at the lesion–bone marrow interface, generates pixel-level uncertainty maps via Monte Carlo Dropout, and validates the association between uncertainty and segmentation error on a multicenter external test set. Furthermore, we conducted physician validation experiments using a within-subject crossover design to systematically evaluate how uncertainty assistance affects clinician trust, review efficiency, and perceived segmentation quality, thereby providing evidence-based support for precision surgical planning and trustworthy AI-assisted clinical decision-making in spinal tuberculosis.

## 2 Materials and methods

This study was a retrospective multicenter validation approved by the ethics committees of the three hospitals, with a waiver of informed consent. The original dataset comprised 300 patients with spinal tuberculosis who underwent preoperative sagittal fat-suppressed T2-weighted MRI. Data were collected from Ningxia Medical University General Hospital (Center 1, *n* = 219), Pingluo People’s Hospital (Center 2, *n* = 43), and Zhoukou Central Hospital (Center 3, *n* = 38). After systematic quality control, 90 cases were excluded, leaving 210 for analysis: 160 from Center 1 for five-fold cross-validation, and 50 (Center 2, *n* = 20; Center 3, *n* = 30) as an external independent test set.

### 2.1 Dataset construction and preprocessing

Preoperative sagittal fat-suppressed T2-weighted MR images were exported from the PACS system in DICOM format. A radiologist with over five years of musculoskeletal imaging experience delineated slice-by-slice along the outer margin of hyperintense lesions to construct 3D ground-truth masks, excluding normal bone marrow edema bands. Images and masks were converted to lossless PNG slices for 2D network input. Two resident physicians independently reviewed all cases in a blinded manner, excluding 31 with mismatched image–mask slice numbers, 34 with severe metal artifacts, 18 with masks containing < 20 foreground pixels, and 7 duplicates. The exclusion of 18 minimal-lesion cases may have artificially inflated segmentation accuracy by removing small or early-stage lesions; the final cohort therefore likely represents a less challenging distribution than unscreened clinical practice.

A strict patient-level partitioning was applied: Center 1 data were used for five-fold cross-validation (approximately 128 training/32 validation cases per fold), while Centers 2 and 3 formed the external test set (*n* = 50), which was not involved in training or hyperparameter tuning. Preprocessing comprised: (1) uniform cropping or zero-padding to 512 × 512; (2) Z-score normalization based on each fold’s training set; (3) for patients with > 15 slices, retention of the central lesion slice and three slices above and below (7 slices total), or maximum available slices if the lesion was near the sequence edge; and (4) extraction of pixel spacing from DICOM headers for spatial metric conversion. Online augmentation during training comprised random horizontal flipping (*p* = 0.5), rotation within ±10° (*p* = 0.3), elastic deformation (*p* = 0.3, sigma = 10, alpha = 100), and gamma correction (*p* = 0.3, range 0.7–1.3).

### 2.2 Deep learning models and training strategy

All models adopted a 2D architecture with an input size of 512 × 512. Baseline model A was a 2D nnU-Net v2 full-resolution configuration. The automatically inferred topology comprised an encoder with 6 stages (initial features: 32, kernel: 3×3, U-Net block depth: 2), instance normalization, and leaky ReLU activation (negative slope 1e-2). The decoder mirrored the encoder with equivalent block structure and trilinear upsampling. Comparative models included classical 2D U-Net (Ronneberger et al., 2015) (B1), attention-enhanced 2D Attention U-Net (Oktay et al., 2018) (B2), and Transformer-CNN hybrid 2D TransUNet (Chen et al., 2021) (B3). The improved model C introduced two innovations based on nnU-Net: (1) Boundary Loss, combined with Dice Loss at a 0.5:0.5 weighting ratio, directly penalizing shifts caused by blurred lesion–bone marrow boundaries; and (2) standard nnU-Net dropout (nn.Dropout2d, *p* = 0.3) was inserted after each convolutional block in both the encoder and decoder, prior to activation; during inference, dropout was kept in train mode to enable T = 30 stochastic forward passes for MC Dropout-based uncertainty quantification.

All five models were independently trained on each fold using the AdamW optimizer (initial learning rate 1 × 10^−4^, weight decay 1 × 10^−5^), cosine annealing decay, and early stopping (patience 30 epochs, monitoring validation Dice). Training was performed on an NVIDIA RTX 3090 GPU with batch size 4; each fold required approximately 5.5 hours (median 210 epochs, range 180–280). For the external test set, uncertainty quantification used a five-fold model ensemble: each fold-specific model performed T = 30 stochastic forward passes, and the mean probability across all 150 predictions (5 folds × 30 passes) served as the final ensemble output. Predictive entropy was computed from pixel-wise probability distributions across all stochastic predictions. Average inference time was 4.2 ± 0.3 seconds per case for a single fold model performing T = 30 passes.

### 2.3 Uncertainty quantification and error association

To assess probabilistic calibration beyond rank correlation, Expected Calibration Error (ECE) and Brier score were computed on the external test set. For ECE, predicted uncertainties were binned into 10 equal-width intervals; ECE was calculated as the weighted average of absolute differences between mean predicted uncertainty and observed pixel-level error rate across bins. The Brier score was computed as the mean squared difference between predicted foreground probability and binary ground-truth label at the pixel level. Logistic regression of case-level uncertainty against binary unreliable-segmentation status was performed to quantify systematic over- or under-confidence, reporting calibration slope and intercept with 95% confidence intervals.

Predictive entropy was calculated for all PNG slices of the 50 external test cases and normalized to 0–1. Uncertainty was stratified into low, medium, and high tertiles based on case-level mean values, and actual pixel-level error rates were calculated for each group. Spearman rank correlation assessed the association between uncertainty and segmentation error. Case-level Dice < 0.80 was defined as unreliable segmentation, referencing the consensus on clinically acceptable minimum overlap from segmentation challenges (ISLES, BraTS) while accounting for inherent difficulty at the blurred lesion–bone marrow edema interface (Ahmed et al., 2024; Gui et al., 2025; Ranjbarzadeh et al., 2024; de Boisredon d'Assier et al., 2024).

The uncertainty threshold was determined via internal cross-validation: on the validation sets of each fold, case-level mean predictive entropy was used as the test variable and unreliable segmentation status as the binary criterion; the maximum Youden index point was calculated per fold, and the median of the five fold-specific thresholds (0.52) was adopted as the preset threshold. This threshold is model-specific and dataset-specific: it was derived from the described 2D nnU-Net architecture with boundary-aware loss and MC Dropout (*p* = 0.3), applied to fat-suppressed T2-weighted MRI with the stated preprocessing and intensity normalization. Direct application to other MRI sequences, scanner vendors, or network architectures without internal recalibration is not warranted. This threshold was not optimized on the external test set but was directly applied to calculate sensitivity, specificity, PPV, and NPV for identifying unreliable segmentation.

### 2.4 Physician validation experiment

Thirty cases were sampled from the external test set using stratified random sampling (10 each with high, medium, and low Dice quality), using a within-subject crossover design. Each physician independently evaluated all 30 cases: 15 were randomly assigned to Group A (control: original image + AI segmentation) and 15 to Group B (experimental: additional uncertainty heatmap overlay). Cases were presented in random order across sessions separated by a washout interval of ≥7 days. The two groups were balanced for lesion segment, size, and center origin.

Nine physicians (3 residents, 3 attending physicians, and 3 associate chief physicians) assessed segmentation quality (Likert 5-point scale), trust scores (1–5 points), and review time (seconds). This included 5 cases of obvious AI failure (Dice < 0.70) to evaluate uncertainty map alert accuracy for erroneous regions.

### 2.5 Statistical analysis

Segmentation performance was evaluated using case-level Dice, IoU, 95% Hausdorff distance (HD95), average surface distance (ASD), sensitivity, and precision, reported as mean ± standard deviation. A single pixel-level ROC curve was generated by aggregating all pixels from the external test set, and AUC was calculated. Inter-model AUC comparisons used the DeLong test; inter-model Dice differences used paired Wilcoxon tests.

For physician validation, a linear mixed-effects model (LMM) was used with fixed effects for group, seniority, and their interaction, and a random intercept for physician; the likelihood ratio test evaluated the main effect of group. Seniority subgroup analysis introduced a group × seniority interaction term, with Wald *χ^2^* tests assessing heterogeneity of uncertainty assistance effects across seniority levels. Pairwise within-subgroup comparisons employed Bonferroni correction (α = 0.017). Inter-physician agreement was evaluated using ICC (two-way random, absolute agreement) and Fleiss’ Kappa. Ordered trends across uncertainty tertiles were compared using the Jonckheere–Terpstra test. For the primary physician-validation analysis (main effects of uncertainty assistance on trust scores, quality scores, and review time), a two-sided *P* < 0.05 was considered significant. For seniority subgroup comparisons, to correct for multiple comparisons across three seniority levels, a Bonferroni-corrected significance level of α = 0.017 (0.05/3) was adopted. All other secondary analyses used *P* < 0.05 unless otherwise specified. Analyses were performed using Python 3.9 (PyTorch 2.0, scikit-learn 1.3) and R 4.3.

## 3 Results

### 3.1 Patient baseline characteristics and data quality

**Figure 1:**
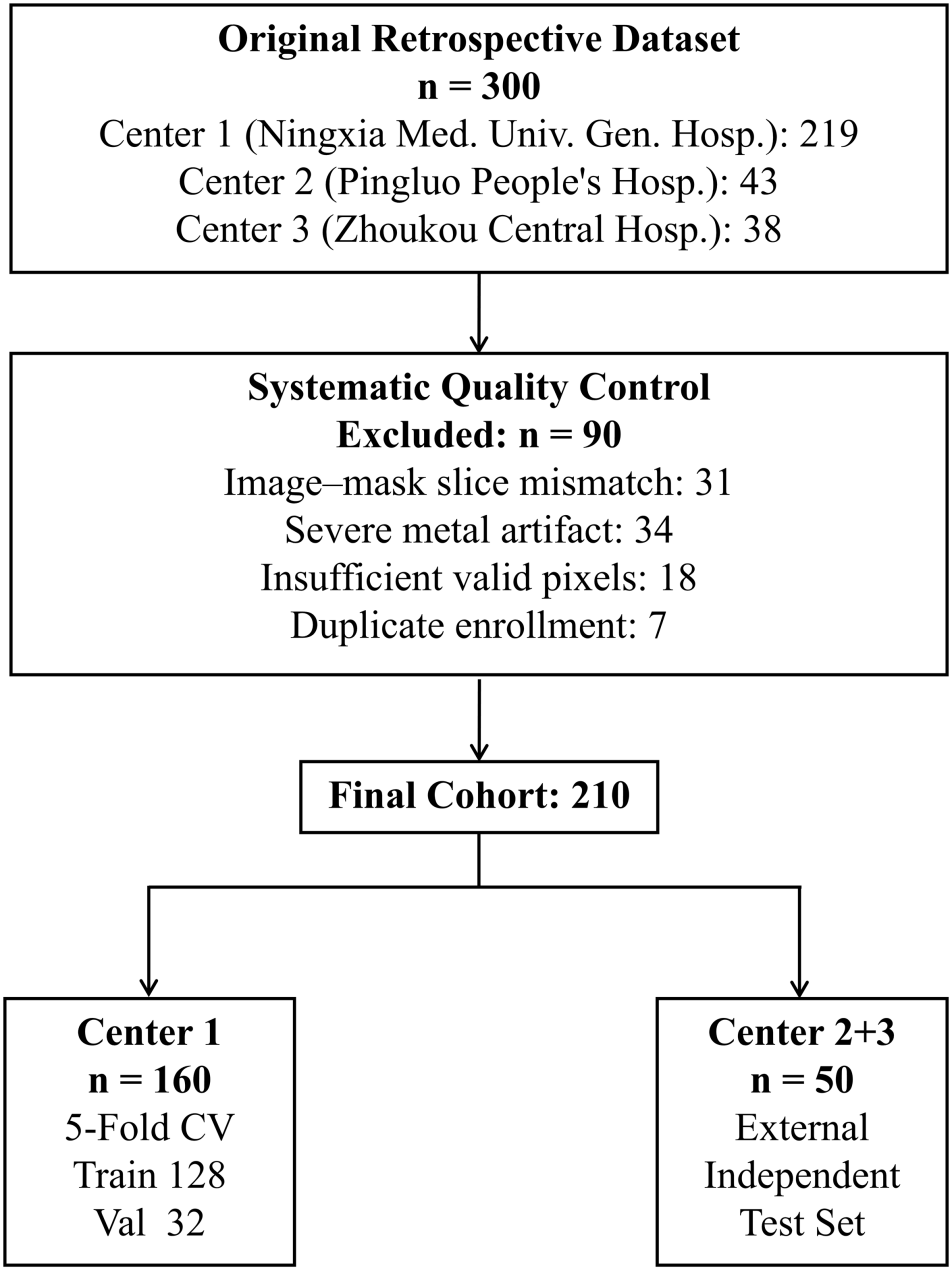
Study workflow and dataset partitioning.

The original retrospective dataset comprised 300 patients with spinal tuberculosis who underwent sagittal fat-suppressed T2-weighted MR imaging, sourced from three centers: Ningxia Medical University General Hospital (Center 1), Pingluo People’s Hospital (Center 2), and Zhoukou Central Hospital (Center 3). After systematic data quality control, 90 cases were excluded (31 with mismatched image–mask slice numbers, 34 with metal artifacts, 18 with masks containing < 20 foreground (lesion) pixels, and 7 duplicate enrollments), leaving 210 cases for subsequent analysis. Among these, 160 cases from Center 1 underwent five-fold cross-validation, while 20 cases from Center 2 and 30 cases from Center 3 jointly constituted the external independent test set (*n* = 50). Patient enrollment, data cleaning, dataset partitioning, and the overall modeling workflow are detailed in **Figure 1**. Baseline characteristics are summarized in **Table 1**. The two groups were well balanced and comparable in age, sex, lesion segment distribution, mean slice number, and number of involved vertebrae (all *P* > 0.05), with only erythrocyte sedimentation rate (ESR) showing a statistically significant difference (*P* = 0.041).

**Table 1:** Baseline characteristics of the study population.

Characteristic	Training/Validation set (n = 160)	External test set (n = 50)	P-value
Age, years	48.2 ± 14.5	49.1 ± 15.2	0.712
Sex			0.876
Male	92 (57.5)	28 (56.0)	
Female	68 (42.5)	22 (44.0)	
BMI, kg/m²	21.8 ± 3.2	22.1 ± 3.5	0.612
Disease duration, months	8.4 ± 6.2	7.9 ± 5.8	0.628
ESR, mm/h	42.6 ± 28.3	55.1 ± 30.1	0.041
CRP, mg/L	28.4 ± 32.1	26.8 ± 29.5	0.742
Involved segments			0.991
Cervical	18 (11.2)	6 (12.0)	
Thoracic	62 (38.8)	19 (38.0)	
Lumbar	80 (50.0)	25 (50.0)	
Vertebrae involved, n	2.4 ± 1.1	2.3 ± 0.9	0.582
Slices per patient, n	7.2 ± 2.8	7.5 ± 3.1	0.512
Paravertebral abscess	124 (77.5)	38 (76.0)	0.832
Epidural abscess	45 (28.1)	14 (28.0)	0.991

Data are presented as mean ± standard deviation or n (%). ESR, erythrocyte sedimentation rate; CRP, C-reactive protein. P-values were derived from the independent-samples t-test or chi-square test, as appropriate. The external test set comprised 20 cases from Pingluo People’s Hospital and 30 cases from Zhoukou Central Hospital.

However, because eleven baseline variables were compared simultaneously, a Bonferroni-corrected threshold of α = 0.0045 (0.05/11) is required to control the family-wise error rate. Under this stringent criterion, the ESR difference is no longer statistically significant, and it should be interpreted as a chance finding rather than evidence of meaningful cross-center heterogeneity. The 90 excluded cases (30% of the initial cohort) were predominantly those with severe metal artifacts or minimal lesion burden. The systematic removal of cases with insufficient valid pixels (*n* = 18) may have enriched the final cohort for larger, more conspicuous lesions, which should be considered when extrapolating the segmentation performance to unscreened clinical populations.

### 3.2 Deep learning model segmentation performance

**Figure 2:**
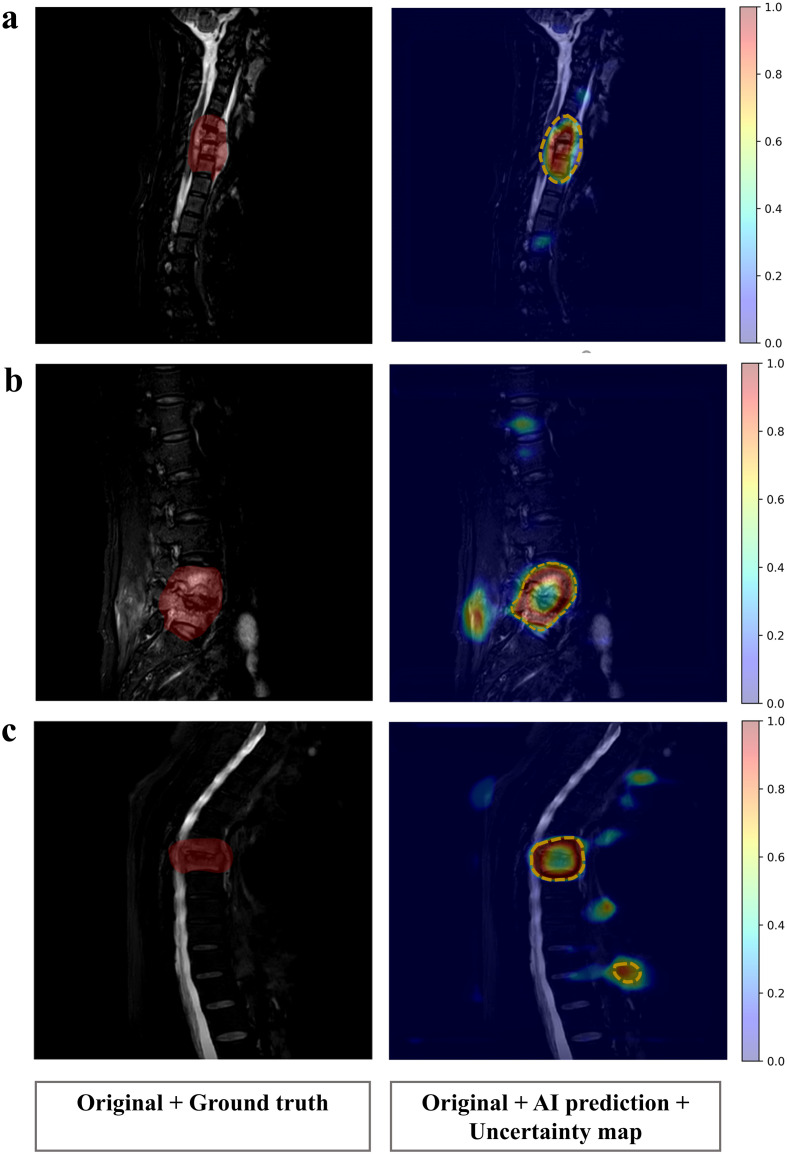
Representative cases of AI segmentation with predictive uncertainty on fat-suppressed T2-weighted MRI in spinal tuberculosis. Ground truth lesions are rendered as semi-transparent red regions, AI predictions as orange-yellow dashed contours, and uncertainty as a jet heatmap. **(a)** Cervical lesion with successful segmentation: the AI contour is highly concordant with the ground truth, and the uncertainty map shows focused activation within the lesion, reflecting confident localization. **(b)** L5–S1 lesion with uncertainty-guided alert: although the AI segmentation is largely consistent with the ground truth, the uncertainty map highlights a small hyperintense zone posterior to the L5–S1 vertebral bodies, prompting the clinician to inspect the paravertebral soft-tissue space for potential infectious extension. **(c)** Segmentation failure with uncertainty flagging: the ground truth annotates a single lesion, whereas the AI incorrectly delineates an additional false-positive focus; the corresponding uncertainty map displays multiple moderate-to-high signal areas, providing a visual cue for physician verification.

The segmentation performance of the five models on five-fold cross-validation and the external test set is detailed in **Table 2**. On the external test set (*n* = 50), improved model C achieved the optimal segmentation performance, with a Dice similarity coefficient of 0.858 ± 0.081, IoU of 0.751 ± 0.052, sensitivity of 0.891 ± 0.038, and precision of 0.834 ± 0.044, all significantly outperforming baseline model A (Dice: 0.841 ± 0.045, *P* = 0.012) and comparative models B1 (0.809 ± 0.052, *P* < 0.001), B2 (0.827 ± 0.048, *P* = 0.003), and B3 (0.815 ± 0.051, *P* = 0.008). Pixel-level ROC analysis further demonstrated that model C achieved an AUC of 0.912, significantly higher than model A (0.894, DeLong test *P* = 0.021), B1 (0.868, *P* < 0.001), B2 (0.883, *P* = 0.009), and B3 (0.876, *P* = 0.005), confirming its comprehensive advantage in lesion-background discrimination. Moreover, model C exhibited the lowest 95% Hausdorff distance and average surface distance among all five models, indicating that boundary-aware loss effectively improved segmentation accuracy in blurred boundary regions between lesions and normal bone marrow edema. **Figure 2** illustrates three representative cases demonstrating the interaction between uncertainty and segmentation. It is noteworthy that improved model C exhibited greater case-level variability (Dice SD = 0.081) than the baseline and comparative models (SD = 0.045–0.052). This heterogeneity indicates that while model C achieved the highest average accuracy, its performance was less uniform across individual patients. *Post-hoc* analysis revealed that the increased variance was driven primarily by a subset of difficult cases characterized by extremely blurred lesion–bone marrow interfaces or small lesion burden; in these cases, model C’s Dice coefficients dropped substantially, whereas in straightforward cases its performance was markedly superior. Importantly, the uncertainty quantification framework successfully identified the majority of these under-performing cases: 14 of the 16 high-uncertainty cases fell within the lowest Dice tertile, suggesting that the elevated variance is clinically manageable when paired with uncertainty-based triage.

**Table 2:** Segmentation performance of deep learning models.

Performance	A: 2D nnU-Net	B1: 2D U-Net	B2: 2D attention U-Net	B3: 2D TransUNet	C: nnU-Net + boundary loss + MC dropout
5-Fold CV Dice	0.864 ± 0.015	0.833 ± 0.020	0.850 ± 0.018	0.840 ± 0.019	0.876 ± 0.014
5-Fold CV IoU	0.760 ± 0.018	0.714 ± 0.025	0.737 ± 0.022	0.723 ± 0.024	0.775 ± 0.016
External Test Dice	0.841 ± 0.045	0.809 ± 0.052	0.827 ± 0.048	0.815 ± 0.051	0.858 ± 0.081
External Test IoU	0.729 ± 0.057	0.683 ± 0.064	0.706 ± 0.059	0.692 ± 0.062	0.751 ± 0.052
External Test AUC	0.894	0.868	0.883	0.876	0.912
External Test HD95 (mm)	4.2 ± 1.6	5.9 ± 2.2	4.7 ± 1.9	5.3 ± 2.0	3.2 ± 1.1
External Test ASD (mm)	1.9 ± 0.7	2.6 ± 1.0	2.1 ± 0.8	2.4 ± 0.9	1.4 ± 0.5
External Test Sensitivity	0.871 ± 0.043	0.844 ± 0.056	0.859 ± 0.050	0.852 ± 0.053	0.891 ± 0.038
External Test Precision	0.820 ± 0.049	0.787 ± 0.062	0.804 ± 0.054	0.791 ± 0.058	0.834 ± 0.044
Wilcoxon P (vs C)	0.012	<0.001	0.003	0.008	Reference
DeLong P (vs C)	0.021	<0.001	0.009	0.005	Reference

CV, cross-validation; IoU, intersection over union; HD95, 95th percentile Hausdorff distance; ASD, average surface distance; AUC, area under the receiver operating characteristic curve at the pixel level. Data for 5-fold CV are presented as mean ± standard deviation across five folds. Data for the external test set are presented as mean ± standard deviation across 50 patients, except for AUC, which represents the pixel-level aggregated area under a single ROC curve across all test pixels. DeLong P-values refer to the comparison of pixel-level AUC against Model C. Wilcoxon P-values refer to the Dice similarity coefficient compared with Model C; DeLong P-values refer to the pixel-level AUC compared with Model C.

### 3.3 Uncertainty quantification and error association analysis

**Figure 3:**
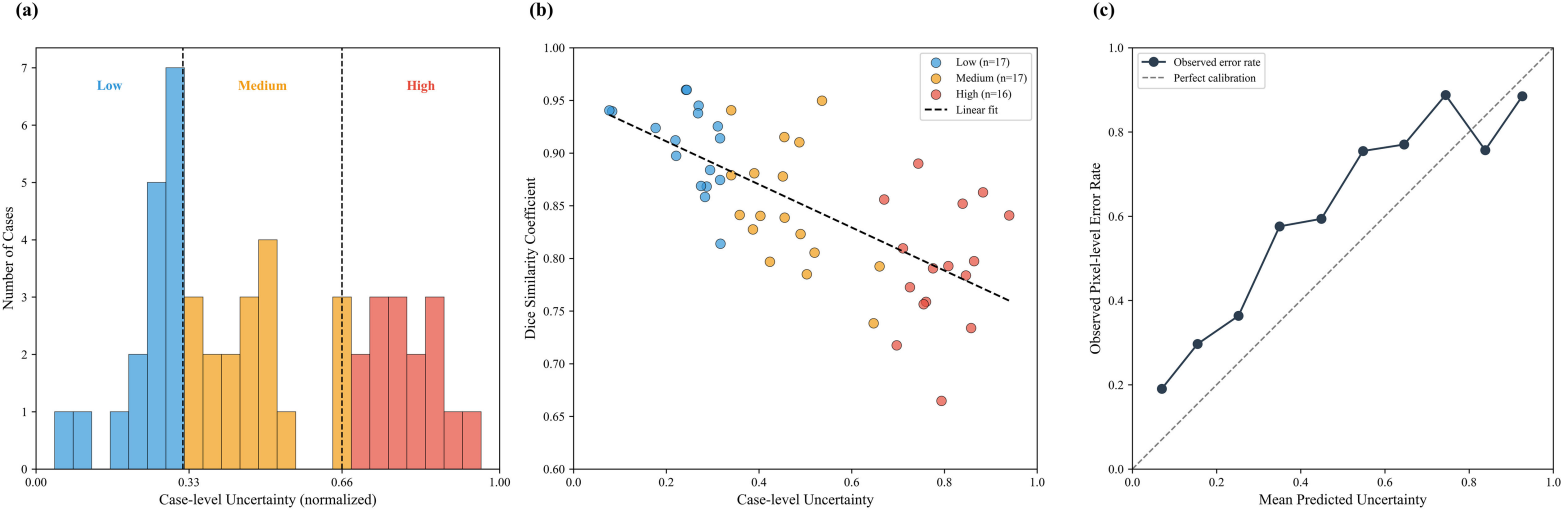
Uncertainty quantification and error association analysis. **(a)** Distribution of case-level uncertainty with tertile thresholds. **(b)** Correlation between uncertainty and Dice similarity. **(c)** Uncertainty-error association plot across 10 equal-width bins, with the diagonal line representing perfect error association.

Uncertainty maps generated by MC Dropout showed a significant positive rank correlation with actual pixel-level error rates on the external test set (Spearman *ρ* = 0.74, 95% CI: 0.63-0.82, *P* < 0.001), indicating a discriminative association. Probabilistic error association was further quantified by an Expected Calibration Error (ECE) of 0.092 and a Brier score of 0.089, indicating moderate but imperfect error association; the predicted uncertainty probabilities deviated from observed error frequencies by approximately 9.2% on average. Logistic regression error association yielded a slope of 0.94 (95% CI: 0.82–1.07) and an intercept of 0.03 (95% CI: –0.08 to 0.14), indicating no significant systematic deviation from perfect error association but confirming the moderate imprecision captured by the ECE. As shown in **Table 3**, after stratification by uncertainty tertiles, the actual pixel-level error rates were 2.1% in the low-uncertainty group, 8.7% in the medium-uncertainty group, and 18.4% in the high-uncertainty group, with statistically significant differences across groups (Jonckheere–Terpstra trend test, *P* < 0.001). Based on five-fold internal validation, the median of the maximum Youden index points across folds (0.52) was adopted as the optimal uncertainty threshold. At this threshold, the sensitivity for identifying unreliable segmentation was 84.3%, specificity was 79.6%, positive predictive value (PPV) was 71.2%, and negative predictive value (NPV) was 89.5%. To assess threshold robustness, we performed a sensitivity analysis across alternative cut-offs (0.40–0.60). As shown in **Table 4**, NPV remained above 85% at all evaluated thresholds, with the most balanced sensitivity–specificity profile observed at the 0.52 cut-off. Clinically, the high NPV (89.5%) is particularly consequential: when the uncertainty map indicates low risk (mean uncertainty < 0.52), clinicians can be confident that approximately 9 out of 10 such cases represent reliable AI segmentations, thereby reducing unnecessary manual review. Conversely, the PPV of 71.2% indicates that among cases flagged as high-uncertainty, roughly 7 in 10 truly represent unreliable segmentation, warranting focused physician scrutiny; however, the remaining ~29% represent false-positive alerts, which is an acceptable trade-off for a safety-oriented screening mechanism. This threshold is model-specific and dataset-specific: it was derived from a 2D nnU-Net architecture augmented with boundary-aware loss and MC Dropout (dropout rate *p* = 0.3), applied to fat-suppressed T2-weighted MRI with the described preprocessing and intensity normalization pipeline. Direct application of this threshold to other MRI sequences, different scanner vendors, or alternative network architectures without internal recalibration is not warranted. **Figure 3** presents the uncertainty distribution histogram, the scatter relationship between case-level mean uncertainty and Dice (R² = 0.42, indicating a moderate linear association), and the uncertainty–error binning plot.

**Table 3:** Segmentation performance stratified by uncertainty tertiles on the external test set.

Uncertainty tertile	Cases, n	Mean case-level uncertainty	Pixel-level error rate, %	Dice	IoU	Sensitivity	Precision
Low (0–0.33)	17	0.22 ± 0.07	2.1	0.911 ± 0.029	0.837 ± 0.046	0.921 ± 0.035	0.905 ± 0.038
Medium (0.33–0.66)	17	0.49 ± 0.08	8.7	0.852 ± 0.037	0.743 ± 0.054	0.881 ± 0.042	0.828 ± 0.048
High (0.66–1.0)	16	0.79 ± 0.09	18.4	0.781 ± 0.053	0.641 ± 0.066	0.842 ± 0.058	0.748 ± 0.062

Uncertainty values were derived from Monte Carlo Dropout (T = 30) and normalized to 0–1. Patients were stratified by mean case-level uncertainty into tertiles. Pixel-level error rate represents the proportion of misclassified pixels relative to the ground truth within each tertile. P for trend across tertiles < 0.001 (Jonckheere–Terpstra test).

**Table 4:** Sensitivity analysis of diagnostic performance across alternative uncertainty thresholds for identifying unreliable segmentation.

Threshold	Sensitivity (%)	Specificity (%)	PPV (%)	NPV (%)
0.40	91.2	68.4	62.1	93.2
0.45	88.1	74.2	66.3	91.5
0.50	85.6	77.8	69.8	90.1
0.52	84.3 (62.4–94.5)	79.6 (63.7–90.8)	71.2 (51.8–86.8)	89.5 (72.8–96.3)
0.55	81.9	82.1	73.6	88.2
0.60	76.4	86.8	78.9	85.7

Values in parentheses are 95% confidence intervals for the 0.52 threshold, calculated using the Wilson score interval.

### 3.4 Physician clinical validation results

**Figure 4:**
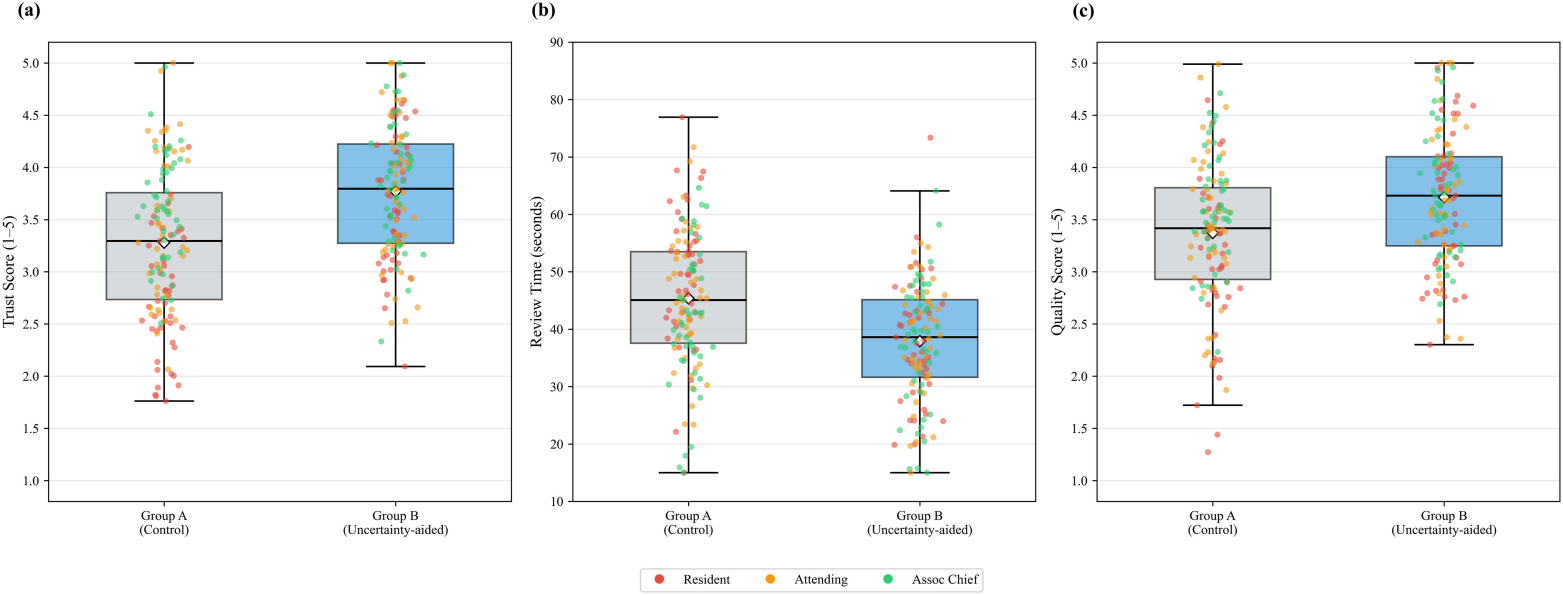
Physician validation: **(a)** trust score, **(b)** review time, and **(c)** quality score. Control **(A)** versus uncertainty-assisted **(B)**.

Physician validation results (within-subject crossover design, *n* = 270 independent evaluations from nine physicians) are summarized in **Table 5** and **Figure 4**. Physicians in Group B (uncertainty-assisted) assigned significantly higher trust scores to AI segmentation (3.8 ± 0.7) than those in Group A (control, 3.2 ± 0.8), with a statistically significant main effect of uncertainty assistance (likelihood ratio test *χ^2^* = 16.42, *P* < 0.001, effect size Cohen’s d = 0.80). Review time in Group B was also significantly shorter than in Group A (likelihood ratio test *χ^2^* = 5.62, *P* = 0.018). Subjective segmentation quality scores were 3.4 ± 0.7 in Group A and 3.7 ± 0.6 in Group B, with a statistically significant difference (likelihood ratio test *χ^2^* = 5.32, *P* = 0.021). Inter-physician scoring agreement was good (ICC = 0.88, 95% CI: 0.82–0.93; Fleiss’ Kappa = 0.76).

**Table 5:** Results of the physician validation experiment.

Variable	Group A (Control)	Group B (Uncertainty-assisted)	Adjusted difference (95% CI)	P-value	Effect size
Overall assessments (*n* = 270)					
Trust score (1–5)	3.2 ± 0.8	3.8 ± 0.7	0.60 (0.42 to 0.78)	< 0.001	d = 0.80
Quality score (1–5)	3.4 ± 0.7	3.7 ± 0.6	0.30 (0.05 to 0.55)	0.021	d = 0.46
Review time, s	45.2 ± 12.3	38.6 ± 10.5	−6.6 (−12.1 to −1.1)	0.018	d = 0.58
Trust score by seniority					
Resident physician (90 evaluations)	2.8 ± 0.6	3.7 ± 0.7	0.90 (0.30 to 1.50)	0.006	d = 1.38
Attending physician (90 evaluations)	3.3 ± 0.7	4.0 ± 0.6	0.70 (0.20 to 1.20)	0.008	d = 1.08
Associate Chief Physician (90 evaluations)	3.5 ± 0.6	3.9 ± 0.5	0.40 (0.02 to 0.78)	0.041	d = 0.73

Group A, original MRI + AI contour; Group B, original MRI + AI contour + uncertainty map. Data are mean ± SD. P-values from LMM; effect sizes, Cohen’s d. ICC = 0.88; Fleiss’ Kappa = 0.76. Alert accuracy: 80.0% (4/5). Nine physicians (3 per seniority) evaluated 30 cases in a crossover design (washout ≥7 days). Group × seniority interaction: χ^2^ = 8.42, P = 0.015. Bonferroni α = 0.017.

**Figure 5:**
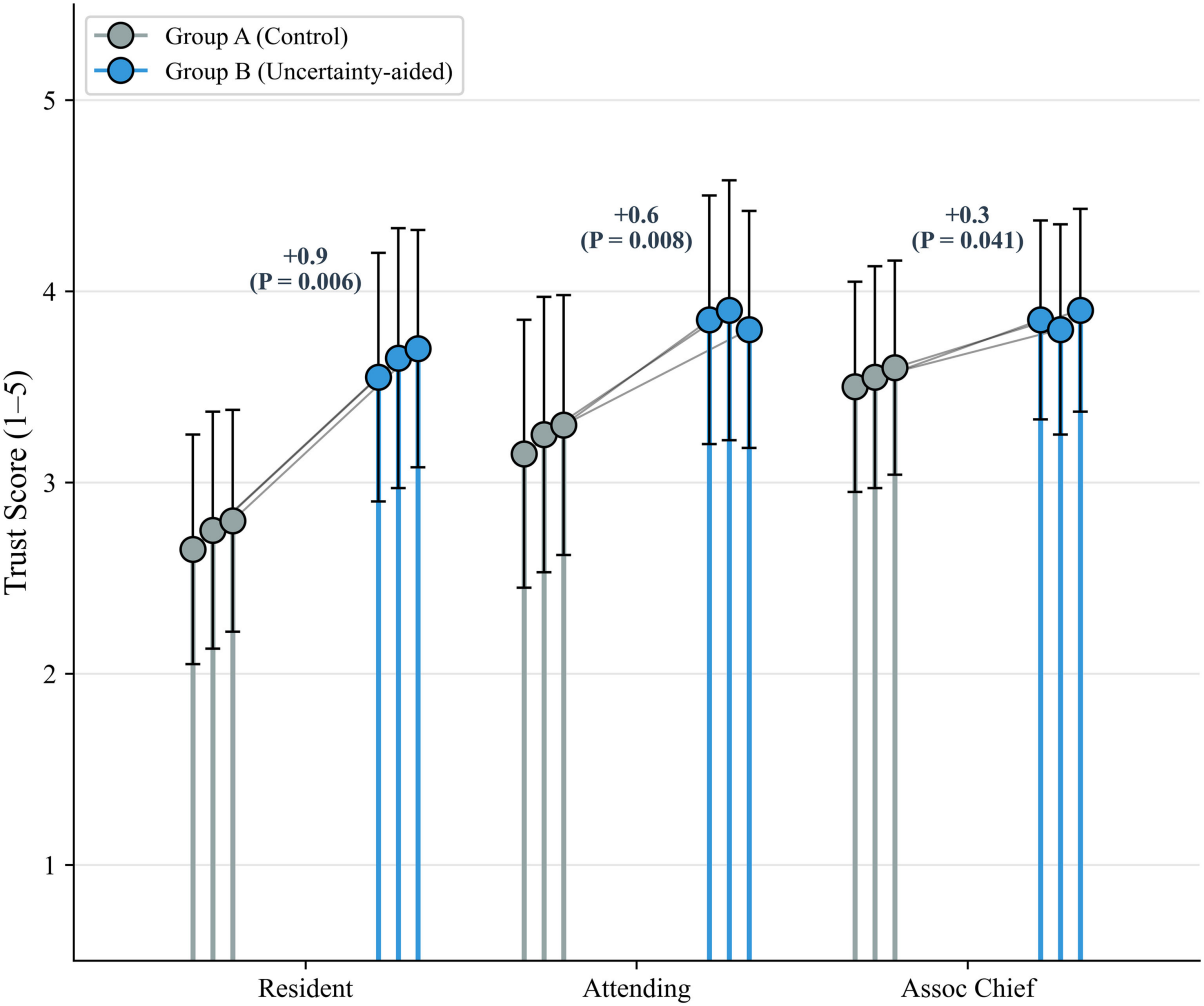
Trust score improvement by physician seniority. Group A (control) versus Group B (uncertainty-assisted). Each data point represents the mean score for one physician; error bars indicate standard deviation. The group × seniority interaction was statistically significant (Wald *χ^2^* = 8.42, *P* = 0.015). Att, attending physician; Res, resident physician; Assoc, associate chief physician.

Seniority subgroup analysis revealed that uncertainty assistance produced the greatest improvement in trust scores among resident physicians (+0.9 points, *P* = 0.006), followed by attending physicians (+0.7 points, *P* = 0.008); the difference for associate chief physicians was smaller (+0.4 points, *P* = 0.041) and did not reach the Bonferroni-corrected threshold (α = 0.017). The group × seniority interaction was statistically significant (Wald *χ^2^* = 8.42, *P* = 0.015), indicating that the magnitude of uncertainty assistance differed across seniority levels. Among the 5 cases of obvious AI failure (Dice < 0.70, including false positives, false negatives, and boundary drift), the uncertainty map achieved an alert accuracy of 80.0% (4/5) for erroneous regions. Seniority subgroup analysis results are detailed in **Figure 5**.

## 4 Discussion

This study constructed an uncertainty-guided deep learning segmentation framework for automated delineation of spinal tuberculosis lesions on fat-suppressed T2-weighted MRI and systematically validated it on a multicenter dataset. Specifically, we introduced boundary-aware loss and Monte Carlo Dropout into the nnU-Net architecture to form improved model C, which demonstrated optimal segmentation performance on five-fold cross-validation and an external independent test set of 50 cases, achieving a Dice similarity coefficient of 0.858 and a pixel-level AUC of 0.912, significantly outperforming baseline nnU-Net and comparative models including U-Net, Attention U-Net, and TransUNet. More importantly, this study evaluated not only segmentation accuracy but also the clinical value of uncertainty quantification: predictive entropy generated by Monte Carlo Dropout showed a significant positive correlation with pixel-level error rates (Spearman *ρ* = 0.74), and the uncertainty threshold of 0.52, determined via internal cross-validation, achieved a sensitivity of 84.3% and a negative predictive value of 89.5% for identifying unreliable segmentation on the external test set. Furthermore, a within-subject crossover physician validation experiment demonstrated that overlaying uncertainty heatmaps significantly enhanced physicians’ trust in AI segmentation (Cohen’s d = 0.80), shortened case review time, and produced the greatest benefit among resident physicians with lower seniority. Collectively, this study provides a relatively complete evidence chain-from technical performance, uncertainty quantification, to clinical usability—for AI-assisted precision segmentation of spinal tuberculosis lesions.

Currently, the application of deep learning in spinal tuberculosis imaging analysis remains in its early stages; existing literature has focused predominantly on lesion detection or classification tasks (Li et al., 2022; Liu et al., 2025; Duan et al., 2023), with limited research addressing precise lesion segmentation. Mo et al (Mo et al., 2026). conducted a multicenter deep learning diagnostic study based on CT bone-window gradient attention mechanisms, enrolling 1,027 patients and employing U-Net to segment vertebrae before feeding them into an improved ResNet50 network to construct an end-to-end spinal tuberculosis diagnostic model. This model demonstrated excellent performance in internal validation (AUC = 0.920, accuracy = 0.874) and showed cross-institutional generalization capability across three external test sets (AUC = 0.866–0.941). The study innovatively utilized CT bone-window gradient features to enhance recognition of trabecular microfractures and calcified margins, offering novel insights for imaging-based AI diagnosis of spinal tuberculosis. However, the model exhibited limited adaptability to heterogeneous data, did not integrate multimodal information such as MRI, and remains to be further developed regarding precise lesion segmentation and uncertainty quantification. Chen et al (Chen et al., 2024). constructed a deep learning model based on conventional MRI sequences to differentiate tuberculous spondylitis (TS) from brucellar spondylitis (BS). This study enrolled 383 patients and employed four CNN architectures—VGG19, ResNet18, VGG16, and DenseNet121—for single-sequence and multi-sequence fusion modeling, finding that the VGG19 model based on three-sequence probability fusion performed optimally (AUC = 0.973, accuracy = 0.921), significantly outperforming two radiologists of different seniority (AUC = 0.804 and 0.882, respectively). This work confirmed the added value of multi-sequence information fusion in differentiating spinal infectious diseases, yet the model focused solely on disease classification without addressing precise lesion segmentation, uncertainty quantification, or external independent validation, leaving room for further investigation regarding clinical translation safety. In the field of uncertainty quantification, although Monte Carlo Dropout has been validated in brain tumor and ischemic stroke lesion segmentation (Zhou et al., 2026; Inamdar et al., 2026; Lilhore et al., 2025; Murase et al., 2025), studies combining it with boundary-aware loss optimization for application to spinal infectious diseases remain scarce. The strengths of this study are manifested in three aspects. First, a rigorous multicenter external validation strategy was adopted: the external test set of 50 cases (20 from Pingluo People’s Hospital and 30 from Zhoukou Central Hospital) was completely independent of the training and hyperparameter tuning processes, effectively evaluating the model’s cross-institutional generalization capability. Second, a within-subject crossover validation experiment conforming to evidence-based medicine standards was designed, controlling for carryover effects through randomized case presentation order and a washout interval of ≥7 days, thereby conferring high internal validity to the physician validation results. Third, uncertainty information was transformed from a “technical parameter” into a “clinical decision-support tool,” directly empowering physicians to identify and correct AI errors through visualized heatmaps, aligning with the ongoing paradigm shift in medical AI from “performance-driven” to “trust-driven” research.

Several key points warrant discussion. FS-T2WI was selected because fat suppression maximally highlights hyperintense lesion edema and inflammatory infiltration, with high sensitivity for early vertebral destruction, paravertebral abscesses, and epidural abscesses. Although T1-weighted and contrast-enhanced T1 sequences provide superior anatomical detail and abscess wall assessment, future multi-sequence fusion may further improve accuracy and uncertainty reliability. Joint boundary-aware and Dice Loss optimization significantly improved segmentation at the lesion–bone marrow edema interface, evidenced by model C’s lowest HD95 (3.2 mm) and ASD (1.4 mm). Blurred boundaries are inherent to spinal tuberculosis (Compagnone et al., 2023; Pande and Babhulkar, 2002; Jevtic, 2004; Balériaux and Neugroschl, 2004; De Vuyst et al., 2003); traditional Dice Loss insufficiently penalizes boundary pixels, whereas boundary-aware loss explicitly measures contour-to-ground-truth distance, mitigating this problem. The 0.52 threshold was derived via internal cross-validation without external-test-set optimization, avoiding data leakage; however, it is model- and dataset-specific, requiring independent recalibration for other sequences or scanners. Clinically, cases below this threshold may proceed to automated reporting, whereas those exceeding it trigger mandatory manual review by senior specialists. Specifically, low-uncertainty cases (< 0.52) may proceed to automated volumetric measurement and preoperative planning, whereas high-uncertainty cases (≥ 0.52) automatically trigger a mandatory manual-review flag, suspending automated report generation until senior confirmation is obtained. Uncertainty assistance improved trust scores most among residents (+0.9, *P* = 0.006), followed by attending physicians (+0.7, *P* = 0.008), with a smaller, Bonferroni-non-significant improvement for associate chiefs (+0.4, *P* = 0.041). The group × seniority interaction was significant (Wald *χ*^2^ = 8.42, *P* = 0.015), consistent with the hypothesis that uncertainty information is more valuable for less experienced practitioners. Nevertheless, with only three physicians per stratum, individual heterogeneity cannot be disentangled from a true seniority effect; this gradient should be regarded as hypothesis-generating and requires multicenter validation. Finally, uncertainty maps localized erroneous regions with 80.0% accuracy in 5 obvious AI failure cases, enabling clinicians to focus on suspicious areas rather than blindly trusting outputs.

This study has the following limitations. First, as a retrospective study, data were sourced from the PACS systems of three hospitals, carrying inherent risks of selection and information bias; the exclusion of 90 cases (30%), including 18 with minimal lesion burden, may have artificially inflated segmentation accuracy by removing the most ambiguous or smallest lesions. The external test set comprised only 50 cases, a relatively limited sample size that may have insufficient statistical power to detect subtle between-group differences. Moreover, a nominally significant baseline difference in ESR levels was observed (*P* = 0.041); however, this lost significance after Bonferroni correction for multiple baseline comparisons and does not provide convincing evidence of systematic cross-center heterogeneity. Second, ground-truth masks were delineated by a single radiologist, introducing the risk of individual bias at blurred lesion boundaries. Nevertheless, two findings support the robustness of our evaluation: (1) the improved model achieved the lowest HD95 (3.2 mm) and ASD (1.4 mm) among all five models, indicating effective boundary drift reduction even against a single expert boundary; and (2) uncertainty maps correlated significantly with pixel-level error (Spearman *ρ* = 0.74), demonstrating an internal consistency independent of the reference standard’s absolute correctness. Future work should employ multi-reader delineation and consensus-based standards. Third, this study employed 2D slice-level input without fully exploiting the three-dimensional spatial context of MRI; future work may explore 3D nnU-Net or 2.5D architectures to improve modeling of lesion spatial continuity. Additionally, this study was based solely on FS-T2WI without integrating T1-weighted, contrast-enhanced T1, or other functional imaging information; multi-sequence fusion is expected to further improve segmentation accuracy in blurred boundary regions. Fourth, the physician validation did not include a pure control group viewing only original images without AI assistance, precluding the disentanglement of the dual effects of AI segmentation itself versus uncertainty overlay, thereby limiting the strength of causal inference regarding the specific incremental value of uncertainty visualization. Fifth, although the physician validation was expanded to nine physicians, this remains a modest sample for random-effects estimation, and the variance components retain wide confidence intervals. While the fixed-effects estimates remain informative, the exact P-values should be interpreted with caution, and the conclusions regarding clinician behavior require replication in larger, multi-institutional physician cohorts. Looking forward, researchers may deepen this work in the following directions: (1) conducting prospective multicenter studies with expanded external validation sample sizes and inclusion of data from different MRI vendors; (2) systematically comparing Monte Carlo Dropout against alternative uncertainty estimation strategies such as Deep Ensemble and Evidential Deep Learning; and (3) embedding the uncertainty-guided segmentation framework into clinical PACS workflows to conduct real-world utility assessments, exploring its translational value in preoperative planning, treatment monitoring, and surgical navigation.

## 5 Conclusion

This study developed and validated an uncertainty-guided framework for spinal tuberculosis segmentation on FS-T2WI. The improved model achieved optimal accuracy, while Monte Carlo Dropout uncertainty maps identified unreliable predictions. Physician validation confirmed that uncertainty overlays enhanced clinician trust and reduced review time, particularly for less senior physicians. This framework provides a clinical decision-support tool combining high precision and interpretability for precision preoperative planning in spinal tuberculosis.

## Data Availability

The raw data supporting the conclusions of this article will be made available by the authors, without undue reservation.
